# A Surgical Case of Synchronous Double Cancer of the Pancreatic Tail and the Distal Bile Duct

**DOI:** 10.70352/scrj.cr.25-0028

**Published:** 2025-06-25

**Authors:** Atomu Suzuki, Yoshinari Maeda, Yusuke Nishio, Taiki Kijima, Yoshinori Kitamura, Seiichirou Ando, Tatsuhito Yamamoto

**Affiliations:** Department of Surgery, Tsushimi Hospital, Hagi, Yamaguchi, Japan

**Keywords:** double cancer, bile duct cancer, pancreatic cancer

## Abstract

**INTRODUCTION:**

In recent years, there have been many reports of cases of double cancer. This is due to improvements in diagnostic techniques and treatment methods for cancer and the extension of average life expectancy. However, there are few reports of bile duct cancer and pancreatic cancer occurring together.

**CASE PRESENTATION:**

The patient was a woman in her 60s who presented to our hospital with jaundice. CT revealed a 30-mm hypovascular mass lesion in the pancreatic head and a similar mass lesion in the pancreatic tail. Endoscopic retrograde cholangiopancreatography (ERCP) showed stenosis of the distal bile duct and pancreatic duct (in the pancreatic tail) without evidence of pancreaticobiliary maljunction. Cytological examination of pancreatic juice revealed Class V adenocarcinoma, while endoscopic ultrasound-guided fine-needle aspiration (EUS-FNA) of the pancreatic tail suggested Class III adenocarcinoma. Based on these findings, a diagnosis of double cancer involving the pancreatic head and pancreatic tail was made. The patient underwent neoadjuvant chemotherapy with five cycles of gemcitabine (1200 mg) and nab-paclitaxel (150 mg). Following chemotherapy, she underwent subtotal stomach-preserving total pancreatectomy with left adrenalectomy and splenectomy. Pathological examination of the resected specimen revealed a 2 × 1.5 cm thickened wall in the distal bile duct associated with bile duct stenosis. In addition, a 3.2 × 1.8 cm nodular lesion was identified in the pancreatic tail, which was not contiguous with the thickened wall of the distal bile duct. Histopathological analysis demonstrated moderately differentiated tubular adenocarcinomas in the distal bile duct and the pancreatic tail. Given the absence of continuity between the 2 tumors and the differences in their histological features, the case was diagnosed as synchronous double cancer of the bile duct and pancreas. The postoperative course was uneventful, and the patient was discharged home on postoperative day 43. However, 2 years after surgery, multiple liver, lung, and lymph node metastases were detected. The patient passed away later that year.

**CONCLUSIONS:**

We report on the case of a patient with bile duct and pancreatic cancer who underwent surgical resection.

## INTRODUCTION

Currently, advancements in diagnostic techniques and treatments for cancer, along with increased life expectancy, have led to an increasing number of reports of multiple primary cancers. However, reports of synchronous double cancers of the bile duct and pancreas remain rare. Here, we report a case of synchronous double cancer involving the distal bile duct and pancreas, which was treated with surgical resection, along with a brief review of the literature.

## CASE PRESENTATION

### Patient

The patient was a woman 69 years old.

### Chief complaint

The chief complaint was jaundice.

### Past medical history

Past medical history was cerebellar infarction, hypertension, and diabetes mellitus.

### Family history

Family medical history was unremarkable.

### History of present illness

The patient had been experiencing itchy skin and loss of appetite for 1 month before presenting at our hospital. She sought medical attention at our hospital after noticing jaundice.

### Relevant physical exam

Height: 145 cm, Weight: 35 kg, BMI: 16.6.

The abdomen was flat and soft, with no palpable masses.

Blood tests revealed a total bilirubin (T-Bil) of 9.65 mg/dL, indicating jaundice. Additionally, elevated levels of liver and biliary enzymes were observed.

Tumor markers were also elevated: CEA 15.4 ng/mL, CA19-9 <2.00 U/mL, DUPAN-2 4400 U/mL, Span-1 130 U/mL.

### Abdominal CT

**Fig. 1:**
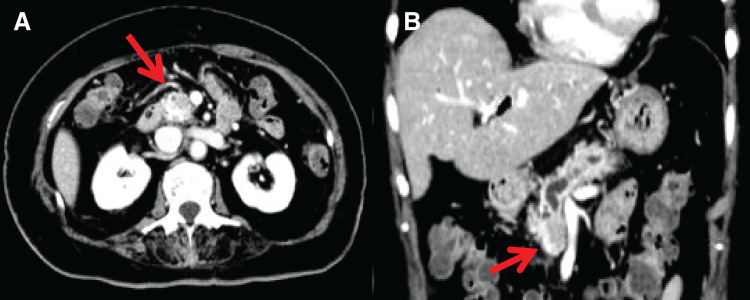
(**A**, **B**) Abdominal CT (pancreatic head). A hypovascular mass lesion was observed in the pancreatic head, partially in contact with the portal vein.

**Fig. 2:**
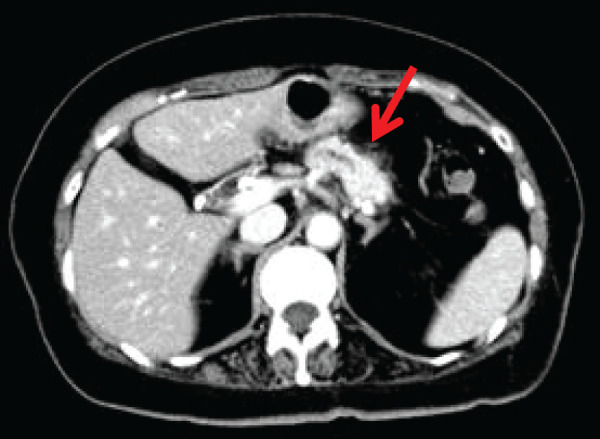
Abdominal CT (pancreatic tail). Disruption of the main pancreatic duct and mass formation were observed in the pancreatic tail.

A hypovascular mass lesion was observed in the pancreatic head. The tumor was in contact with the portal vein at approximately 45 degrees, but no obvious invasion was observed. The tumor size was 24 mm (**Fig. 1A**, **1B**). A 30-mm mass was also identified in the pancreatic tail with the fat stranding sign (**Fig. 2**). Endoscopic ultrasound-guided fine-needle aspiration (EUS-FNA) revealed Class III findings suggestive of adenocarcinoma.

### Endoscopic retrograde cholangiopancreatography

**Fig. 3:**
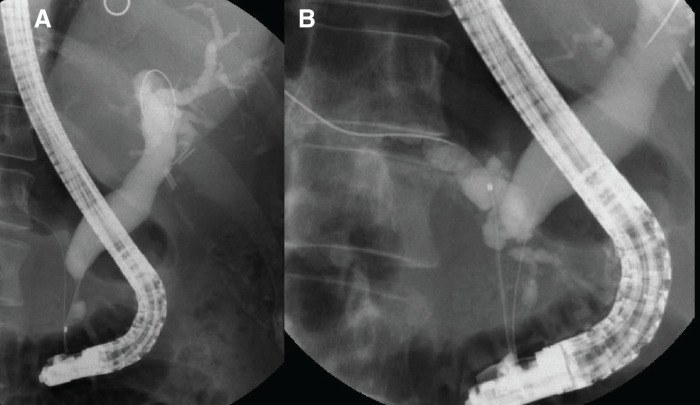
(**A**, **B**) Endoscopic retrograde cholangiopancreatography. Stenosis of the distal bile duct and pancreatic duct was observed, with no evidence of pancreaticobiliary maljunction.

Stenosis of the distal bile duct and pancreatic duct was observed, with no evidence of pancreaticobiliary maljunction (**Fig. 3A**, **3B**).

Cytological examination of pancreatic juice revealed Class V adenocarcinoma.

### PET-CT

**Fig. 4:**
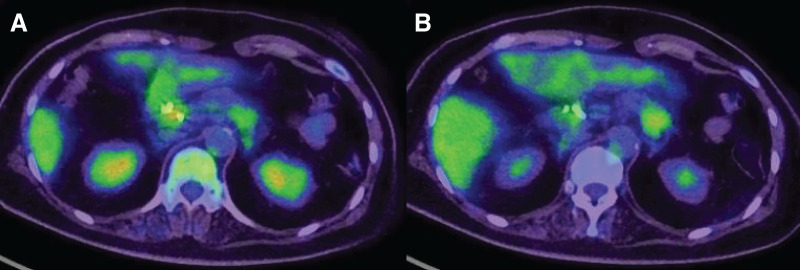
(**A**) PET. (**B**) CT. A mass lesion in the pancreatic head exhibited an SUV max of 3.8, while a mass lesion in the pancreatic tail showed an SUV max of 3.6.

A mass lesion in the pancreatic head exhibited an SUV max of 3.8, while a mass lesion in the pancreatic tail showed an SUV max of 3.6 (**Fig. 4A**, **4B**).

### Preoperative diagnosis

Preoperatively, the case was considered to be synchronous double cancers of the pancreatic head and the pancreatic tail.

1. Ph, TS2 (24 mm), infiltrative type, cT3, cCH1, cDU0, cS0, cRP1, cPV0, cA0, cPL0, cOO0, cN0, cM0(P0, H0), CYX

TNM classification: cStage IIA (Resectable)

2. Pt, TS2 (30 mm), infiltrative type, cT2, cCH0, cDU0, cS0, cRP1, cPV0, cA0, cPL0, cOO0, cN0, cM0(P0, H0), CYX

TNM classification: cStage IB (Resectable)

### Preoperative treatment

Preoperative chemotherapy with gemcitabine (1200 mg) and nab-paclitaxel (150 mg) was administered for a total of 2 courses to treat the synchronous double cancers of the pancreatic head and pancreatic tail. The tumor marker levels showed the following trends: CEA (ng/mL): 15.4 → 11.7, DUPAN-2 (U/mL): 4400 → 5000, SPAN-1 (U/mL): 130 → 77.

No change in tumor size was observed before and after chemotherapy (stable disease: SD).

As a complication of chemotherapy, Grade 3 neutropenia was noted.

### Surgical findings

A midline upper abdominal incision was made to open the abdomen. No evident non-curative factors were observed, such as peritoneal dissemination or liver metastases. Subtotal stomach-preserving total pancreatectomy with left adrenalectomy and splenectomy was performed. Based on preoperative imaging findings suggestive of posterior invasion, the left adrenal gland was completely resected.

### Pathological findings

**Fig. 5:**
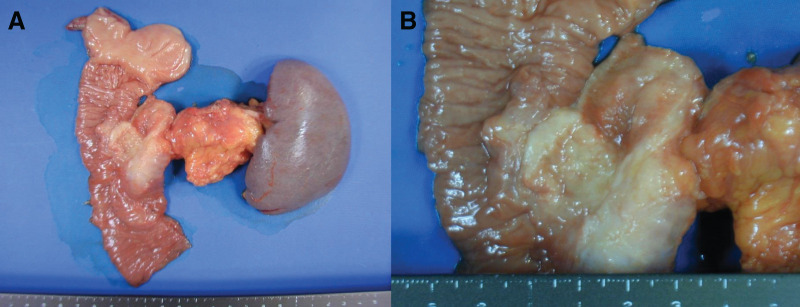
(**A**, **B**) Pathological findings. On cross-sections of the resected specimen, a 2 × 1.5 cm area of wall thickening was observed in the distal bile duct, along with stenosis of the distal bile duct. Additionally, a 3.2 × 1.8 cm nodular lesion was identified in the pancreatic tail, which was not contiguous with the wall thickening of the distal bile duct.

**Fig. 6:**
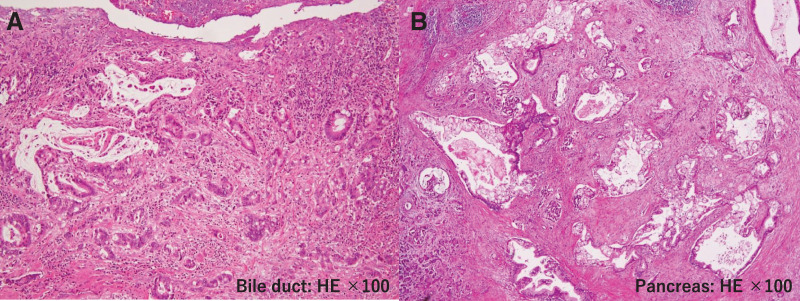
(**A**, **B**) Pathological findings (H.E., ×100). Both the bile duct and the pancreatic lesion were moderately differentiated tubular adenocarcinomas. The tumor in the pancreatic tail was predominantly composed of cells with relatively abundant cytoplasm and mucin production. Marked degenerative changes were observed in the tumor cells, presumably as a result of preoperative chemotherapy. In the pancreatic head, the tumor consisted mainly of small cells with a high nuclear-to-cytoplasmic ratio. While some areas showed irregular small glandular structures, the majority exhibited infiltrative growth in trabecular patterns or formed small nests composed of a few cells. Degenerative changes were also present in these tumor cells, although they were less prominent than those seen in the pancreatic tail. In some regions, the epithelium was denuded, and no dysplastic changes were identified in the adjacent bile duct epithelium. However, as the tumor was centered around the bile duct, the findings were not inconsistent with cholangiocarcinoma.

On cross-sections of the resected specimen, a 2 × 1.5 cm area of wall thickening was observed in the distal bile duct, along with stenosis of the distal bile duct. Additionally, a 3.2 × 1.8 cm nodular lesion was identified in the pancreatic tail, which was not contiguous with the wall thickening of the distal bile duct (**Fig. 5A**, **5B**). Although both the bile duct and the pancreatic lesion were moderately differentiated tubular adenocarcinomas, the histological features differed, and no continuity was observed between the tumor cells of the two lesions. The tumor in the pancreatic tail was predominantly composed of cells with relatively abundant cytoplasm and mucin production. Degenerated tumor cells, presumably due to treatment effects, were conspicuous. In the pancreatic head, the tumor was composed of small cells with a high nuclear-to-cytoplasmic ratio, forming irregular small glandular structures in some areas. However, the majority exhibited infiltrative growth in trabecular patterns or as small nests consisting of a few cells. Although degenerative changes were also observed in these tumor cells, they were less prominent than those in the pancreatic tail lesion. In some areas, the epithelium was denuded, making it difficult to identify dysplastic changes in the adjacent bile duct epithelium. Nevertheless, as the tumor was centered around the bile duct, the diagnosis of cholangiocarcinoma remains plausible. Therefore, this case was diagnosed as synchronous double cancers of the bile duct and pancreas (**Fig. 6A**, **6B**).

Bile duct: Tubular adenocarcinoma, tub2

Bd, tub2, pT3a, INFb, ly1, v0, ne1, HM0, pN0

Pancreas: Invasive ductal carcinoma, tub2 > tub1

Pt, tub2, ypT3, ypCH0, ypDU0, ypS1, ypRP1, ypPV0, ypA0, ypPL0, ypOO0, ypPCM0, ypBCM0, ypDPM0, pN0

### Postoperative course

Although the postoperative course was free of infectious complications, the patient experienced difficulty in glycemic control and was discharged home on POD 43. The patient remained recurrence-free and maintained a good general condition for 2 years following surgery. However, during the second postoperative year, multiple metastases to the liver, lungs, and lymph nodes were identified. Following the administration of one course of gemcitabine plus nab-paclitaxel (GEM + nabPTX), the patient experienced anorexia and deterioration in general condition, requiring hospitalization and intravenous supportive treatment. Subsequently, the treatment was switched to gemcitabine monotherapy, and one course was administered. Following the onset of diarrhea, fatigue, and edema, chemotherapy was discontinued, and the patient was transitioned to palliative care. The patient passed away later that year due to the primary disease.

## DISCUSSION

In recent years, the incidence of synchronous double cancers in the gastrointestinal tract has been increasing, likely due to the aging population’s advancements in treatments for malignant diseases and the marked progress of diagnostic imaging technology.^1)^ However, reports of synchronous double cancers involving the bile duct and pancreas are rare, possibly because the prognosis of both diseases is generally poor. The widely accepted definition of synchronous double cancers is based on the criteria established by Warren and Gates in 1932.^2)^ According to these criteria presented by Warren and Gates, synchronous double cancers are defined by the following three conditions: 1) Each tumor must exhibit malignant histology, 2) Each tumor must be located at a distinct site, separate from the other, and 3) One tumor must not be a metastasis of the other. Furthermore, Moertel et al.^3)^ classified synchronous double cancers into synchronous and metachronous types based on an interval of 6 months or less for synchronous and more than 6 months for metachronous cancers. The incidence of multiple primary cancers involving pancreatic cancer has been reported to be 3.2% in resected pancreatic cancer cases and 5.6% in autopsy cases.^4,5)^

According to reports from PubMed and the Japan Medical Abstracts Society (Ichushi database) in Japan, there were only seven reported cases of synchronous double cancers involving pancreatic cancer and bile duct cancer, including our case^4–9)^ (**Table 1**). The majority of initial symptoms were jaundice. However, only three cases were diagnosed as synchronous double cancers preoperatively. In the remaining cases, surgery was performed based on a preoperative diagnosis of either pancreatic cancer or bile duct cancer, and synchronous double cancers were confirmed in the resected specimens postoperatively. As a result, most cases involved tumors in the distal bile duct for the biliary lesions and the pancreatic head region for the pancreatic lesions. It was considered difficult to preoperatively distinguish between the biliary and pancreatic lesions as separate entities. Furthermore, none of the reported cases exhibited pancreaticobiliary maljunction. While the role of pancreaticobiliary maljunction in the development of pancreatic cancer remains unclear, it has been suggested that the reflux of pancreatic juice into the biliary tract, caused by maljunction, contributes to carcinogenesis in biliary tract cancers.^10)^ In the seven cases reviewed, no pancreaticobiliary maljunction was observed, suggesting that maljunction is minimally involved in the pathogenesis of synchronous double cancers of pancreatic and bile duct origin. Regarding overall prognosis, three out of six cases resulted in death within 1 year,^1,7,10–12)^ indicating a poor prognosis. Considering that both pancreatic cancer and bile duct cancer individually have poor prognoses, synchronous double cancer involving these two malignancies may be associated with an even worse prognosis. However, due to the limited number of reported cases, this remains a matter of debate.

**Table 1:** Reported cases of double cancers occurring in bile duct and pancreas

No	Author	Age	Gender	Complain	Diagnosis of double cancer	NAC	Pancreas (stage)	Biliary duct (stage)	R	Prognosis
1	Yoshii^5)^	71	M	Jaundice	Post operation	–	Ph, poor (unknown)	Bd pap (unknown)	R0	Unknown
2	Akiyama^6)^	68	F	Jaundice	Pre operation	–	Phb, well (T3N1stageIII)	Bd pap (T1N0 stageI)	R2	Dead (8M)
3	Kitagawa^7)^	77	F	Weight loss	Post operation	–	Ph, moderatelly (T3N1stageIII)	Bd tub1 (T1N0stageI)	R0	Dead (7M)
4	Sato^8)^	74	M	Jaundice	Pre operation	–	Ph, mucinous (unknown)	Bd tub3 (T4N2stageIVa)	Unknown	Dead (8M)
5	Kato^9)^	78	M	Jaundice	Post operation	–	Ph, well (T2N3stageIVB)	Bd sig (T4N2stageIVa)	R0	Alive (14M)
6	Hosokawa^4)^	73	M	Abdominal pain	Pre operation	–	Ph, moderatelly (T3N0stageIII)	Bp tub2 (T4N0stageIVa)	R0	Alive (84M)
7	Our Case	69	F	Jaundice	Post operation	GEM+nab PTX	Pt, moderately (T3N0ypstageIII)	Bd tub2 (T3N0stageIIB)	R0	Dead (2Y)

Various factors, including curative resection, lymph node metastasis, perineural invasion, and histological differentiation, have been reported as independent prognostic factors for bile duct and pancreatic cancers.^11–15)^ Curative resection and lymph node metastasis are considered the most significant predictive factors for both bile duct and pancreatic cancers. In our case, the combination of preoperative chemotherapy and curative surgical resection likely contributed to 2 years of recurrence-free survival.

Regarding the diagnosis of synchronous double cancers in our case, based on the criteria established by Warren & Gates, conditions 1) and 2) were pathologically confirmed. However, complete verification of condition 3) was challenging. Nevertheless, the bile duct tumor and pancreatic tumor, despite both being moderately differentiated tubular adenocarcinomas, exhibited different histological features, leading to the diagnosis of synchronous double cancers. In reports of collision cancers, the importance of immunohistochemical staining has also been highlighted. Hirono et al.^16)^ also reported differences in CK20, MUC2, and p53 expression levels in immunostaining cancers found in the bile duct and Vater’s ampulla. In cases where histological evaluation is challenging or distant metastases cannot be ruled out, it may assist in the diagnosis. We performed immunohistochemical staining using CK20, CK7, p53, MUC1, MUC2, and MUC5AC. Although there were some regional differences, the results were insufficient to draw definitive conclusions based solely on immunohistochemistry. These findings are considered supplementary and should be interpreted as an aid to diagnosis rather than definitive evidence.

## CONCLUSIONS

We experienced a surgically resected case of synchronous double cancers involving the distal bile duct and the pancreas. Preoperative diagnosis and treatment, as well as the selection of an appropriate surgical procedure, are crucial.

## DECLARATIONS

### Funding

Not applicable.

### Authors’ contributions

YM, YN, TK, YK, SA, and TY performed the surgery and postoperative management.

All authors read and approved the final manuscript.

### Availability of data and materials

Not applicable.

### Ethics approval and consent to participate

This work does not require ethical considerations or approval. Informed consent to participate in this study was obtained from the patient.

### Consent for publication

Written informed consent was obtained from the patient for publication of this case report and any accompanying images.

### Competing interests

The authors declare that they have no competing interests.
